# A Conformation-Specific Approach to Native Top-down Mass Spectrometry

**DOI:** 10.1021/jasms.4c00361

**Published:** 2024-10-25

**Authors:** Hannah M. Britt, Aisha Ben-Younis, Nathanael Page, Konstantinos Thalassinos

**Affiliations:** † Institute of Structural and Molecular Biology, University College London, London WC1E 6BT, United Kingdom; ‡ LGC Group, Teddington TW11 0LY, United Kingdom; § Institute of Structural and Molecular Biology, Birkbeck College, London WC1E 7HX, United Kingdom

## Abstract

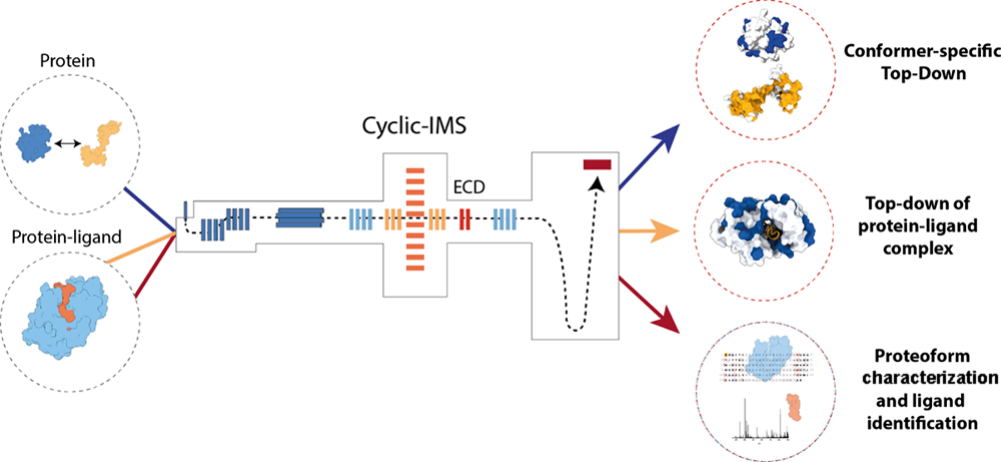

Native top-down mass spectrometry is a powerful approach for characterizing proteoforms and has recently been applied to provide similarly powerful insights into protein conformation. Current approaches, however, are limited such that structural insights can only be obtained for the entire conformational landscape in bulk or without any direct conformational measurement. We report a new ion-mobility-enabled method for performing native top-down MS in a conformation-specific manner. Our approach identified conformation-linked differences in backbone dissociation for the model protein calmodulin, which simultaneously informs upon proteoform variations and provides structural insights. We also illustrate that our method can be applied to protein–ligand complexes, either to identify components or to probe ligand-induced structural changes.

## Introduction

True understanding of biological function at the molecular level requires thorough examination of protein variation resulting from differences in genetics, protein structure or post-translational modifications (PTMs).^1^ Top-down MS (TDMS) is an invaluable tool for the in-depth characterization of these protein variants, termed proteoforms. In TDMS, ion activation is used to dissociate the covalent bonds making up the protein backbone.^2,3^ These covalent bonds break in a predictable fashion, enabling the amino acid sequence of the protein, including modifications to its residues, to be determined from the molecular masses of the observed fragments. Under the right conditions, TDMS can provide almost complete sequence coverage, and therefore comprehensively characterize PTMs, single nucleotide polymorphisms (SNPs), and splice or truncation variants.^4−7^ While cleavage of every inter-residue bond in a protein to enable complete coverage is desirable in characterizing these modifications, it is rarely achieved in practice, with the exception of very small model proteins. This is attributed to the relatively low fragmentation efficiency of protein species, as they get larger and adopt relatively low charge states. As such, considerable effort in recent years has been placed in novel instrumentation and approaches to overcome these limitations in fragmentation efficiency.^2,8−11^ Ignited by these developments, sufficient sequence coverage has been achieved to prove the power of TDMS to identify proteins and characterize proteoforms in a variety of biological areas, including in complex systems such as human biofluids and tissue samples.^12−15^

Historically, to maximize sequence coverage and increase proteoform identification, TDMS has been performed under denaturing conditions, making the protein backbone accessible for fragmentation. Recently, however, native top-down mass spectrometry (nTDMS) has come to the fore, in which proteins are introduced into the mass spectrometer using native sample preparation and soft ionization conditions to retain their solution-state characteristics.^16−19^ The major benefit of nTDMS is that protein–protein and protein–ligand interactions remain intact, and therefore, proteoforms are characterized within this context. While protein fragmentation patterns observed in TDMS have provided exquisite proteoform detail to date, this advent of nTDMS has added an extra dimension to the analysis by enabling attempts to derive structural information from fragmentation patterns.^20−22^ Since their development in the late 1990s, electron-based fragmentation techniques have proved particularly fruitful in this endeavor, with the product ions produced accepted to be sensitive to protein structure.^23−28^ Similar experiments have even been used to successfully map key biological features on the protein backbone such as protein complex interfaces, regions of helicity, and ligand-binding sites.^29−31^ Ultraviolet photodissociation (UVPD) has similarly shown exciting promise for linking protein fragmentation to 3-dimensional structure.^32^ Notable structural insights ranging from characterization of protein–protein interaction and ligand binding regions through to documenting unfolding pathways have been revealed using the UVPD approach.^33−36^ Furthermore, recent attempts have proven successful in linking protein structural moieties to fragmentation patterns obtained from infrared photon-based modalities, alongside collision-based methods previously thought to be unable to report on conformational elements.^20,37^

A key limitation in the use of nTDMS to inform on protein conformation, however, is that the majority of instruments currently used in such studies lack ion mobility (IM) separation and the ability to separate and isolate conformers prior to activation. While this is not an issue for primary structural characterization, it becomes problematic when coexisting conformers exist, as the fragmentation patterns will be a composite of the two conformers rather than a reflection of a single structure. This inability to link top-down information to individual conformers hampers biological interpretation of the data, especially when structural differences are key to protein function or disease pathogenesis. Proteins with multiple conformations that have different activity are treated as a single species, rather than being studied individually for their unique characteristics. Similarly, in the case of protein misfolding, distinctive features of incorrectly folded proteins would be indistinguishable from the correctly folded analog.

Incorporation of Ion Mobility (IM) into the nTDMS workflow is one approach that can provide added value for users looking to link top-down fragmentation data to conformational elements of proteins. IM is able to provide a low resolution readout of protein structure, which if used in sequence with a dissociation method, directly links structure to fragmentation. As such, combining these two powerful methods goes a long way in verifying which structure(s) is present in any given nTDMS experiment, thus further validating structural interpretation of the results. Such workflows are particularly beneficial for proteins thought to be highly disordered or those known to exist in multiple conformations. Similarly, the approach is particularly fruitful in the case of protein misfolding, allowing distinctive features of incorrectly folded proteins to be easily distinguished from those of the correctly folded analogue.

There are several flavors of IM, including drift tube (DTIMS), traveling wave (TWIMS), trapped (TIMS), and field asymmetric waveform (FAIMS), which are described in detail elsewhere, however they all act to separate ions based on their size and shape.^38−41^ For some molecules, including proteins, IM measurements can even be turned into low resolution structural information in the form of collision cross section (CCS) values, which can then be matched to crystals or structures obtained from molecular dynamics experiments.^42−47^ These IM methods have been successfully integrated into a range of MS instrumentation with exciting results, however due to their synergistic time scales the majority of commercial instruments pair IM with time-of-flight (ToF) analyzers.^48−54^ To date, attempts have been made to combine linear IM with TDMS experiments on ToF instruments both pre- and postactivation, and these methods have shown great benefits in distinguishing backbone isomerization and sequence variation, improving sequence coverage, and mapping regions of unfolding or structural variation.^55−59^ While these are impressive achievements, it could be argued that since the matching of fragmentation patterns to single conformers occurs during the postcollection processing rather than the actual MS workflow, attempts to match structural elements directly to conformers could be subject to interference or cross-contamination, introducing a source of unreliability. More recently, new instruments with enhanced IM resolution and tandem IM capabilities have been developed, which can potentially overcome this reliance on processing by enabling single conformer isolation in-instrument.^60−63^ To date, these more advanced IM instruments have been combined with collision, electron and surface-based activation techniques, however, the focus of these studies has been either using dissociation methods pre-IM and using the IM resolution gains to improve sequence coverage, or performing single conformer isolation on denatured structures purely for the purpose of distinguishing isomers.^64−67^ As such, the benefits of these enhanced IM capabilities, particularly the isolation of single conformers, in interpreting native protein fragmentation patterns in the context of protein structures remain unexplored.

**Figure 1:**
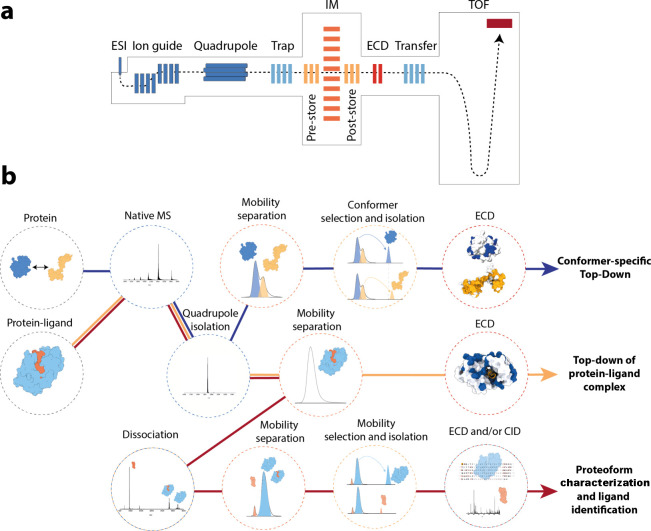
(a) Schematic of the cIM quadrupole time-of-flight instrument used to implement the nIM-TDMS approach. The instrument is based on the commercial cIM platform with postmobility ECD modification. (b) Depending on whether a protein or protein–ligand complex is analyzed, different workflows can be followed. For a protein with more than one conformation, tandem ion mobility can be used to isolate each conformer for ECD fragmentation, thus obtaining conformer-specific top-down data (blue line). For protein–ligand complexes, two workflows exist. ECD fragmentation of the complex, which can reveal structural changes due to ligand binding (orange line), or dissociation of the complex followed by isolation of each component using ion mobility, followed by ECD or CID fragmentation (red line). This workflow allows for identification of the bound ligand.

Here we present a new native ion mobility top-down mass spectrometry (nIM-TDMS) approach that uses tandem IM to selectively isolate single native protein conformers in-instrument for conformation-specific top-down fragmentation using structure-sensitive electron-based fragmentation. In doing so, this method enables a confident structural interpretation of the resulting fragmentation patterns by correlating them directly to specific protein conformational states. The approach was implemented on a cyclic ion mobility (cIM)-enabled quadrupole ToF instrument with postmobility electron capture dissociation (ECD) capabilities, shown in Figure 1a.^60,61^ ECD is an electron-based dissociation method wherein a stream of electrons is released from a filament, resulting in their interaction with the analyte molecule and, in the case of proteins, subsequent backbone cleavage. For readers interested in the fundamental principles and applications of this technique, we recommend consulting a number of excellent reviews.^2,68,69^ ECD was selected as our chosen dissociation method due to its proven track record in mapping fragmentation patterns to structure while retaining protein/ligand interactions. The geometry of this instrument allows nIM-TDMS of proteins (blue line) and their complexes (orange line) through the workflows detailed in Figure 1b. The tandem IM capabilities of the cIM instrument combined with top-down capabilities further enable the characterization of ligands associated with single conformer protein complexes (Figure 1b red line), enabling for the first time to our knowledge MS^n^ capabilities on a quadrupole ToF instrument.

## Methods

### Sample Preparation

Calmodulin from bovine testes was purchased as a lyophilized powder (P1431 Sigma, U.K.) and stored in aliquots at −20 °C as a 40 μM stock solution in 10 mM ammonium acetate. For analysis, stock solution was diluted in 10 mM ammonium acetate to a final concentration of 5 μM. Melittin from honey bee venom (M2272 Sigma, U.K.) and Calmodulin-dependent Protein Kinase II fragment 290–309 (C4926 Sigma, U.K.) were stored as stock solutions at 1 mM in 10 mM ammonium acetate at −20 °C. For all ligand binding experiments, the peptides were added to calmodulin at a final concentration of 7.5 μM, giving a 1:1.5 protein/ligand ratio.

### Mass Spectrometry

Mass spectrometry experiments were performed on a SELECT SERIES Cyclic IMS QToF (Waters Corp., U.K.) fitted with a postmobility ECD modification (e-MSion, U.S.).^60,70,71^ The instrument was operated in sensitivity mode and calibrated using sodium iodide (NaI) to within 1 ppm over the *m*/*z* range 0–8000. Samples were infused into the instrument using nano electrospray (nESI) capillaries prepared in house using a Flaming-Brown P97 micropipette puller, and gold-coated with a Quorum Q150RS sputter coater. Parameters used for analyses were: Capillary Voltage 1.4 kV; Sampling Cone 40 V; Trap Collision Energy 8 V; Transfer Collision Energy 6 V.

### Ion Mobility

Ion mobility experiments were also performed on the ECD-modified SELECT SERIES Cyclic IMS QToF (Waters Corp., U.K.) using the MS parameters detailed above. For these experiments, the instrument was operated in mobility mode with the following parameters: Automatic ADC; 1 Push Per Bin; Racetrack Bias 70 V; Twave Height 28 V; Twave Velocity 375 ms^–1^. The instrument was operated in single pass mode (5 ms separation time) for all separations detailed in this study, since multiple passes were not found to appreciably increase resolution between calmodulin’s mobility peaks (Supporting Information, SI, Figure S3) in line with previous observations of cytochrome C.^61^

For classic native top-down experiments in which no conformer selection was required, the following cyclic control sequence was used: Inject - Separate for 5 ms, and Eject Ions and Acquire. Complete cyclic sequences for all additional experiments are presented in Table S1 within the SI Methods of this manuscript. Further information regarding the geometry of the SELECT SERIES Cyclic IMS QToF (Waters Corp., U.K.) and the preparation of cyclic control sequences for protein analysis, including activation, are described in detail elsewhere.^60,61,72^

### Electron Capture Dissociation

Electron capture dissociation was performed at the postmobility ECD cell (e-MSion, U.S.), with the voltages across the block (L1; L2; LM3; L4; FB; LM5; L6; L7) tuned using reported *c* and *z* ions for calmodulin to give the optimum dissociation while retaining ion transmission. The values used were L1 1.0; L2–30.0; LM3 10.0; L4 12.0; FB 3.4; LM5 8.5; L6–30.0; and L7 1.0. For all experiments the filament current was maintained at 2.3 A. The Transfer Collision Energy was increased to 16 V, providing supplemental activation to reduce nonspecific fragment binding. For calmodulin-peptide complexes the FB was dropped to 2.9 but all other parameters remained the same.

### Data Processing

Native mass spectra and IM data were processed in MassLynx v4.2 (Waters Corp.), and deconvoluted using UniDec v4.3.0.^73^ ECD data were processed using UNIFI (Waters Corp., U.K.). Raw spectra over the *m*/*z* range 200–8000 were deconvoluted for 30 iterations using the BayesSpray module operated in intact protein mode with a ToF resolution of 25 000.^74^ The resulting [M + H]^+^ ion masses were then matched within 20 ppm error to theoretically generated *b*, *y*, *c*, and *z* fragment ions for bovine calmodulin (UniProt P62157) using ProSight Lite.^75^ Reported PTMs for bovine calmodulin, *N*-terminal methionine loss, *N*-terminal acetylation, and K^115^ trimethylation, were also applied to the sequence during matching.^76^

## Results and Discussion

### Analysis of Calmodulin

**Figure 2:**
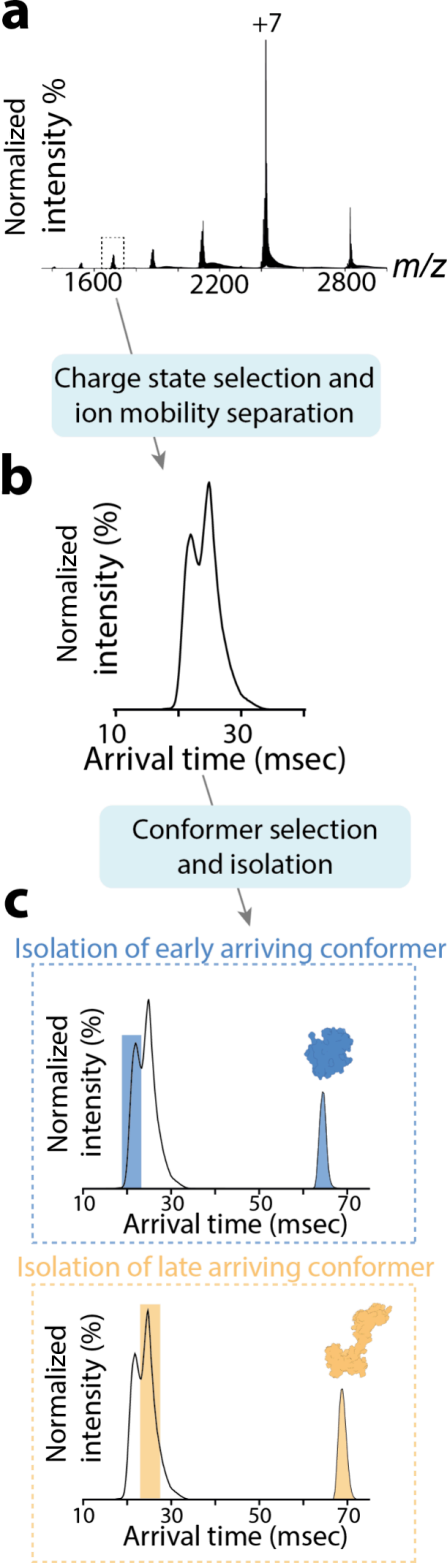
From the native mass spectrum of calmodulin (a), the +10 charge state was selected for subsequent analysis. At least two distinct conformers can be observed in the arrival time distribution (ATD) for this charge state (b), each of which were isolated for ECD fragmentation, here shown as the blue and yellow ATDs for early and late mobility regions, respectively (c).

**Figure 3:**
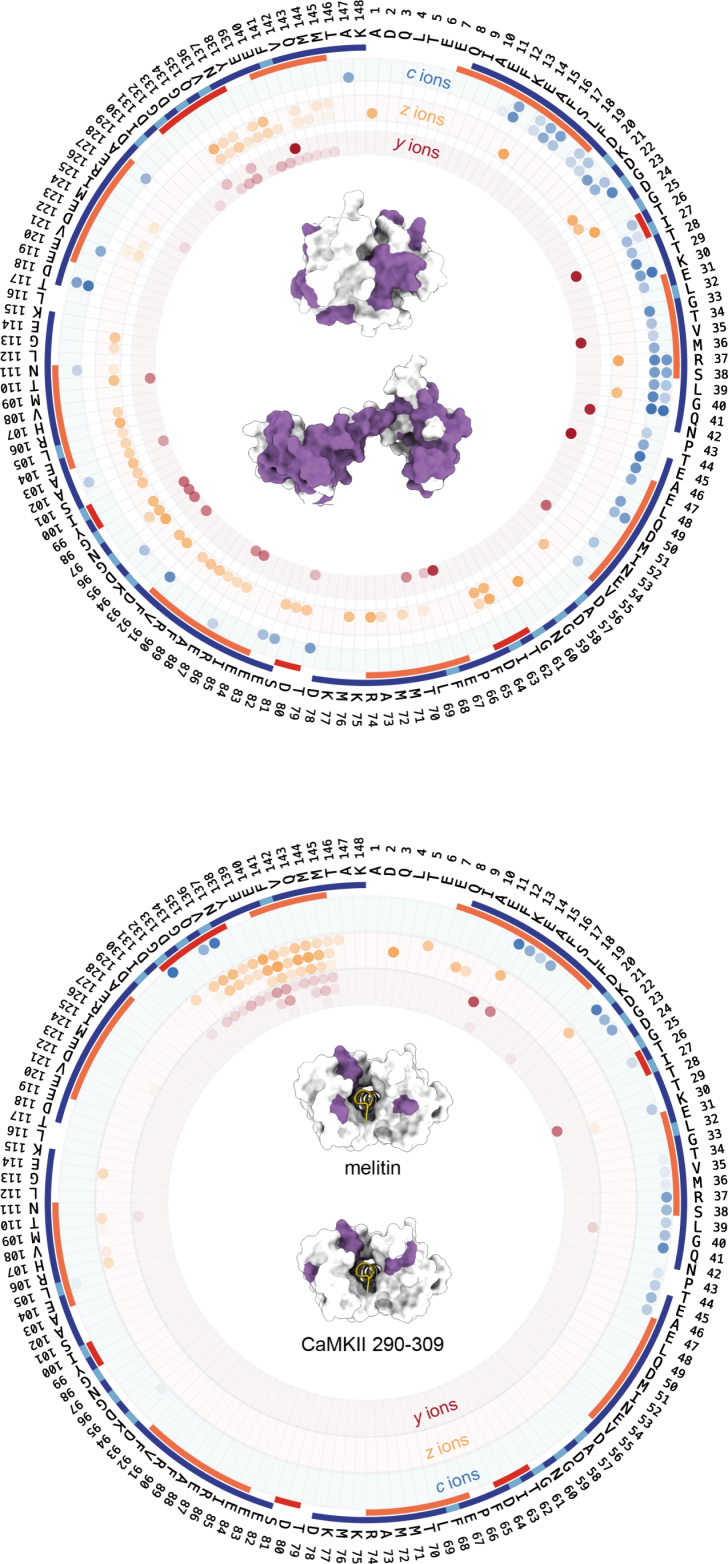
ECD fragmentation data are displayed against the calmodulin sequence in the circular plots above, with colored rectangles indicating different secondary structural elements (alpha helices, orange; beta strands, red; EF-hand, blue; calcium binding sites–cyan). The three main ion fragment series observed (*c*, *z*, and *y* ions) are shown as concentric circle slices. In the upper plot, the outermost circle shows the *c* fragments for the compact and extended conformers, followed by the *z* ions for the compact and extended conformers, and the innermost circle shows the *y* ions for the compact and extended conformers. Each fragment is represented as a colored dot, with the color indicating the normalized fragment ion intensity. In the center of the circle, the PBD structures representing the compact (1PRW) and extended conformers (3CLN) are shown, with fragmentation sites mapped in purple. The lower plot presents equivalent ECD fragmentation data for the apo, melittin, and CaMKII 290–309 bound calmodulin (outer to inner circles, respectively). It should be noted that the apo form here contains fewer fragments than the corresponding data in the upper panel, as a lower charge state (+8) was used. The PDB structure for the canonical calmodulin-ligand complex (1C1M) is shown in the center of the circle along with the fragment sites (purple) obtained from each ligand-bound experiment.

We applied nIM-TDMS to study the protein calmodulin, which undergoes conformational changes upon binding to calcium and small peptides. The protein was electrosprayed from native-like conditions, yielding a charge state distribution from 5+ to 12+, corresponding to a species consistent with calmodulin (theoretical Mw 16791.5 Da). The quadrupole was then used to isolate the 10+ charge state (Figure 2a), which exhibits a mixed calcium-binding occupancy of *n* = 0–5 (SI Figure S1) reflecting the protein’s reported state within its native environment. Due to the resolving power of the quadrupole and the close spacing between calcium-bound calmodulin states, it was not found to be within the capabilities of the instrument to cleanly isolate a distinct calmodulin-Ca^2+^ population, hence the use of a mixed-calcium binding state for these studies. Ion mobility separation of the charge state of 10+ revealed two conformational families, as demonstrated by the arrival time distributions (ATD) in Figure 2b. Following calibration, these species equate to conformers with CCS values of 1539 Å^2^ and 1966 Å^2^, respectively, which are consistent with literature measured and theoretical CCS values for globular (1PRW) and dumbbell (3CLN) calmodulin.^76−79^ Details on the CCS calibration method used can be found in the SI Methods of this manuscript. Using the tandem cIM capabilities, each conformational family was then isolated from the rest of the population within the instrument, as shown in Figure 2c, forming a clean ion population. Individual conformers were then subjected to ECD. The two families exhibited different dissociation patterns, with the extended one showing considerably more fragmentation, made up of the *c* and *z* ions associated with electron-based methods, alongside *y* ions at 67% backbone cleavage, compared to 27% for the compact form (Figure 3 upper panel). Fragmentation of the extended population was spread over a greater proportion of the sequence, while the compact structure’s dissociation was predominantly at the protein termini. Preferential fragmentation at the termini, combined with nonspecific behavior in regards to cleavage between residues, largely correlates with published data regarding the amino acid cleavage preferences by ECD rather than slow-heating methods, providing confidence that the observed variation between conformers is structurally driven rather than a feature of the dissociation mechanism.^80,81^ It should be noted, however, that some contribution to the variation in fragmentation pattern could be attributed to differences in calcium binding state between the two conformations (SI Figure S1), however given the consistency of our observation with literature findings, we believe that this contribution is likely minor and that structural differences drive the observed changes.^58^ Further interrogation of this contribution could be achieved by searching for fragment ions where Ca^2+^ ions remain bound; however, this remains challenging at present due to a dearth of suitable software to identify these highly mobile modifications to fragments. In comparison, performing a similar experiment without conformer selection (SI Figure S2) resulted in a fragmentation pattern corresponding to 63% backbone cleavage, a similar or improved value compared to literature reports for nTDMS of calmodulin, but containing overlapping fragments from both families.^58,82,83^ This highlights the necessity of using a conformation-specific approach to correctly attribute fragmentation findings to the protein structure.

By performing top-down fragmentation on isolated conformational families, we were able to directly link the observed fragmentation patterns to structural elements of the protein. We highlight the bonds that fragmented on the PDB structures most representative of our compact and extended forms of calmodulin in the upper panel of Figure 3. The central region of the protein, specifically residues 60–114, had a greater number of fragmentation sites in the extended conformer than the compact form, particularly *z* and *y* ions. This additional fragmentation can be directly linked to the increased solvent accessibility of the central helix of calmodulin (residues 65–93) in the extended structure, which has previously been reported in covalent labeling-MS studies and solution state structural methods.^84−88^ This observation is consistent with the general trend observed in the data, that higher levels of bond dissociation are directly linked to higher levels of solvent accessibility. Detailed comparison to established methods for characterizing protein solvent accessibility, such as hydrogen–deuterium exchange (HDX), would be helpful in confirming this observation; however, HDX provides insight only into the bulk solution state. As such, the method is not specific enough to provide information directly on single conformations to enable a direct comparison to the nIM-TDMS workflow. Beyond the clear link between solvent accessibility and dissociation propensity, we did not observe a strong correlation between either amount of fragmentation or ion type with the specific secondary-order protein structures, for example there was no observation of the 3–4 residue cleavage pattern sometimes associated with helical structure.^81^ While there are clearly fragmentation differences between some structural regions, for example, the helix between 119 and 127 has minimal fragmentation compared to several *z* and *y* ions in the dual helix-sheet region between 134 and 145, we do not feel that these contrasts are significantly consistent across regions to draw definitive conclusions on secondary structure-specific fragmentation patterns. This observation is consistent with the variation in published literature in this area, it seems that there remain insufficient examples in order to form a universally accepted opinion on the relationship between ECD fragmentation patterns and structural protein regions.

The isolation of specific fragment ions corresponding to each conformation would have also been possible through computational methods such as TWIMExtract.^89^ However, by instead isolating these conformers in-instrument, our method minimizes the risk for spectral contamination. Furthermore, when applying this postcollection processing approach to our own data (SI Figure), we found the differences in fragmentation pattern between conformers to be far less pronounced than when using in-instrument isolation. It should also be noted that isolating a single conformer allows for further experimental investigations to gain deeper insights into its properties before it undergoes ECD fragmentation. For instance, an isolated conformer could be trapped in the gas phase over extended periods of time prior to ECD to study the gas phase annealing phenomena in more detail.

Interestingly, within the conformer-specific regions of differing dissociation is a known calcium-binding motif between residues 93–100. The extended conformer formed *c* (1), *y* (4), and *z* (5) fragments in this region, compared with only one *z* ion in the compact, suggesting that this site could be used for calcium binding within the compact structure but not the more extended form. This hypothesis fits with the PDB structure, literature, and intact mass measurement (SI Figure S3), which suggests the majority of calmodulin has 2–3 calcium occupancy in this state, compared to 3–4 for the compact conformer. Three other known calcium-binding sites (colored cyan in Figure 3) showed similar fragmentation between the two structures, consistent with comparable calcium occupation and their locations away from the solvent-accessible central helix. These sites did show slightly reduced fragmentation compared to surrounding regions, relative to their proximity to the protein termini where high levels of bond dissociation are expected, supporting the idea that within these regions the backbone would be protected from ECD fragmentation by calcium binding.

### Analysis of Calmodulin-Peptide Complexes

Binding of ligands often plays a critical role in modulating a protein’s function. We, therefore, developed two additional workflows, shown in Figure 1b, which enable in-depth interrogation of protein/ligand complexes. The first workflow, nIM-TDMS of a protein–ligand complex (orange line), is comparable to the single-protein approach, with a single conformer of the intact complex isolated, then probed directly by ECD. Application of this experiment is here demonstrated for 1:1 (protein/ligand) complexes of calmodulin with two ligands, melittin and calmodulin-dependent protein kinase II (CaMKII) fragment 290–309 (Figure 3 lower panel). While it has previously been reported that complete calcium occupancy is not a requirement for the binding of these peptide ligands to calmodulin, we found the *n* = 4 calcium-bound state dominated our spectra for both complexes.^90^ As the protein:ligand complexes occupy a lower charge state distribution than the free protein, the species selected for ECD were 8+ rather than 10+, which had a noticeable effect on the fragmentation efficiency, as is expected with electron-based dissociation techniques. It is still possible, however, to identify regions of the protein backbone that show reduced fragmentation in the presence of ligands, for example, the *N*-terminal residues 13–46, residues 106–114, and some *C*-terminal residues from 130 to 146. Mapping these regions onto the canonical calmodulin-peptide structure (PDB 1C1M), Figure 3 lower panel, shows that they all have close proximity or H-bonding to the peptide binding pocket, suggesting that solvent-accessibility reductions and noncovalent interactions drive the reduced fragmentation. A small difference in the dissociation pattern for the two ligand bound species is observed between residue 135–138, which could be attributed to the reduced length of CaMKII peptide (19 aa) compared to melittin (26 aa) altering the solvent accessibility in this region; however, being a short stretch, it is difficult to be completely confident in this conclusion without supporting data.

**Figure 4:**
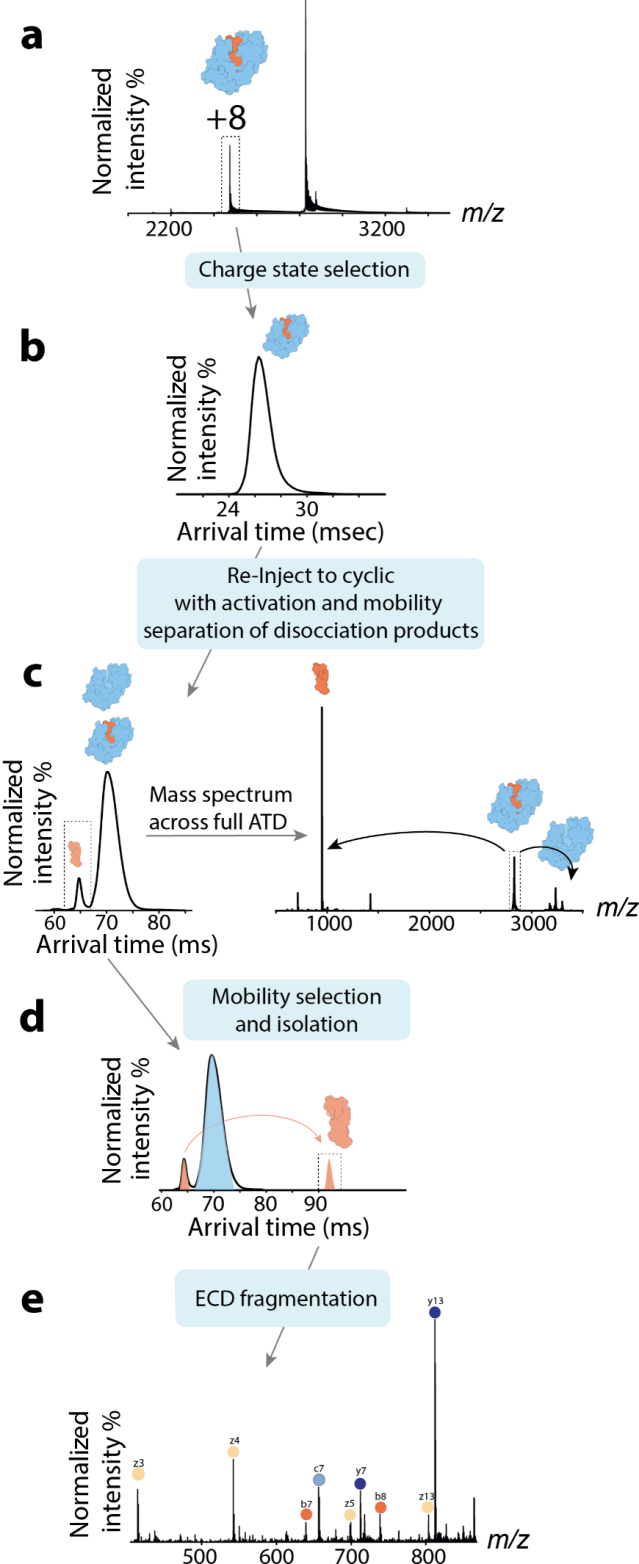
Analysis of calmodulin-ligand complexes using nIM-TDMS. From the native mass spectrum of the calmodulin:melittin (1:1) complex (a), the +8 charge state was quadrupole-isolated and mobility separated, giving a single species ATD (b). The complex was then dissociated into its protein and ligand constituents by increasing the injection energy upon reinjection to the mobility region (c). The ligand (orange) was then mobility selected (d) and subjected to ECD fragmentation, allowing identification of melittin through fragment annotation (e).

In the last nIM-TDMS workflow (Figure 1c red line), it is possible to characterize the ligands that are involved in single-conformation protein/ligand complexes, in an approach similar to that of the native-omics workflow first described by Gault and colleagues, but for the first time using QToF instrumentation.^91^ The workflow for this (Figure 4) involves native introduction of the protein/ligand complex into the mass spectrometer, followed by *m*/*z* selection of a single protein/ligand species. The complex is then mobility separated, and a single conformation of the complex isolated in the prearray store, as demonstrated for a calmodulin-melittin complex in Figure 4b. The complex is then collisionally dissociated by application of activation upon its reinjection from the prearray store into the mobility cell, causing it to break down into its component parts. Tandem IM can then be applied to isolate only the mobility region associated with the peptide ligand, Figure 4d, allowing for ligand identification by top-down MS using either ECD or CID, Figure 4e. In this way, unlike the native-omics approach, in which multistage MS experiments are performed based upon a *m*/*z* isolation, our workflow instead performs species selection based upon mobility, overcoming the MS-in-space limitations traditionally associated with QToF instrumentation. As demonstrated, this mobility selection of specific conformational species works particularly well for peptides such as melittin (Figure 4d), as they occupy significantly different arrival times compared to the free protein or protein/ligand complex. We expect similar behaviors for ligand-types such as peptides, organic molecules, and lipids, given their differing mobility compared to proteins, opening up the door to applying this method for the identification of these ligands within protein complexes. Subsequent fragmentation of the ligand by ECD or CID can then be performed. The application of activation to release the ligand is predicted to negate the relevance of any structural findings from this workflow; however, sufficient coverage is achieved to enable successful identification of melittin from the calmodulin-ligand complex (Figure 4e). Given this level of coverage, we foresee the approach being particularly applicable for identifying unknown ligands from within native complexes, for example endogenous complexes where the specific ligand bound might otherwise not be clear due to lack of previous characterization. As with similar approaches, however, such endeavors would require knowledge of the ligand fragmentation patterns to aid identification, and while these are well reported for peptides and glycans, they become considerably more variable or less informative when wanting to identify small molecules or lipids.

## Conclusions

Using nIM-TDMS we have demonstrated the usefulness of a conformation-specific approach for direct nTDMS analysis of proteins and their complexes. Instead of sampling across the entire conformational landscape or relying on postprocessing extraction methods, we can selectively isolate specific protein conformers within the instrument, which enables us to link the intrinsic structural and proteoform information obtained from nTDMS experiments direcly to specific protein conformations. Our conformation-specific approach is therefore perfectly placed to provide structural insights and component characterization for proteins and their complexes all within the same experiment, making it invaluable in enabling a true biological understanding of systems where proteins exhibit multiple structures and transient or low abundant structural intermediates. We believe that nIM-TDMS has great potential in the structural characterization of traditionally challenging families of proteins and complexes, such as intrinsically disordered proteins, glycoproteins, membrane proteins, and proteins that are prone to misfolding and aggregation. nIM-TDMS can also provide amino acid level, conformation specific data for integrative structural biology workflows, supplying information which is currently underused due to difficulties in obtaining it by classic structural or biophysical methods, but which is required to provide a truly accurate picture of the systems studied.^92^

## Supporting Information Available

Figures detailed in the text and tables detailing the matched fragment ions with error tolerances for each data set discussed in the main text (PDF)
