# Supplementary material for: A Conformation-Specific Approach to Native Top-down Mass Spectrometry
Source: J Am Soc Mass Spectrom. 2024 Oct 25;35(12):3203–13. doi: 10.1021/jasms.4c00361 (PMC11622372; doi:10.1021/jasms.4c00361)
Supplement: Supplementary file 1 — js4c00361_si_001.pdf [file js4c00361_si_001.pdf]

Supplementary Information: A conformation-specific approach to native top-down mass spectrometry

Hannah M. Britt[1], Aisha Ben-Younis[1], Nathanael Page[1,2], Konstantinos Thalassinos[1,3]*

1 Institute of Structural and Molecular Biology, Division of Biosciences, University College London, London, WC1E 6BT, United Kingdom

2 LGC Group, Teddington, TW11 0LY, United Kingdom

3 Institute of Structural and Molecular Biology, Birkbeck College, University of London, London, WC1E 7HX, United Kingdom

* Contact email: k.thalassinos@ucl.ac.uk

SUPPLEMENTARY METHODS

Table S1. Details of the cyclic control sequences that were used for mobility isolation experiments detailed throughout the manuscript:

**Experiment Type**	**Inject**	**Separate**	**Eject Ions**	**Eject to Pre-Store**	**Eject Ions**	**Reinject from Pre-Store**	**Separate**	**Eject Ions & Acquire**
No selection	Yes	5 ms (1 pass)	-	-	-	-	-	Yes
Compact conformer isolation	Yes	5 ms (1 pass)	3.5 ms	2 ms	22.5 ms	No activation	5 ms (1 pass)	Yes
Extended conformer isolation	Yes	5 ms (1 pass)	8 ms	2 ms	18 ms	No activation	5 ms (1 pass)	Yes
Protein-Ligand Complex Dissociation	Yes	5 ms (1 pass)	0 ms	28 ms	0 ms	100 V activation	5 ms (1 pass)	Yes

CCS values were only calculated for the no selection single pass experiments, as at the time of writing debate still exists in the community regarding the best way to CCS calibrate multi-pass and mobility selected cIM data. Calibration was performed using the logarithmic fit method for native proteins (Ruotolo B.T., Benesch J.L.P., Sandercock A.M., Hyung S., and Robinson C.V., Ion mobility–mass spectrometry analysis of large protein complexes. *Nat. Protoc*. 2008, **3 (7)**, 1139-1152. for details). The calibrants used were Ubiquitin (Sigma, UK, U6253), Cytochrome C (Sigma, UK C7752), monomer and dimer β-lactoglobulin (Sigma, UK, L7880), Avidin (Sigma, UK, A9275), and Bovine Serum Albumin (Sigma, UK, P7656), and reference values were obtained from the Bush Calibrant Database (Bush M.F., Hall Z., Giles K., Hoyes J., Robinson C.V., and Ruotolo B.T., Collision Cross Sections of Proteins and Their Complexes: A Calibration Framework and Database for Gas-Phase Structural Biology. *Anal. Chem.*, 2010, **82 (22)**, 9557–9565.)

SUPPLEMENTARY FIGURES

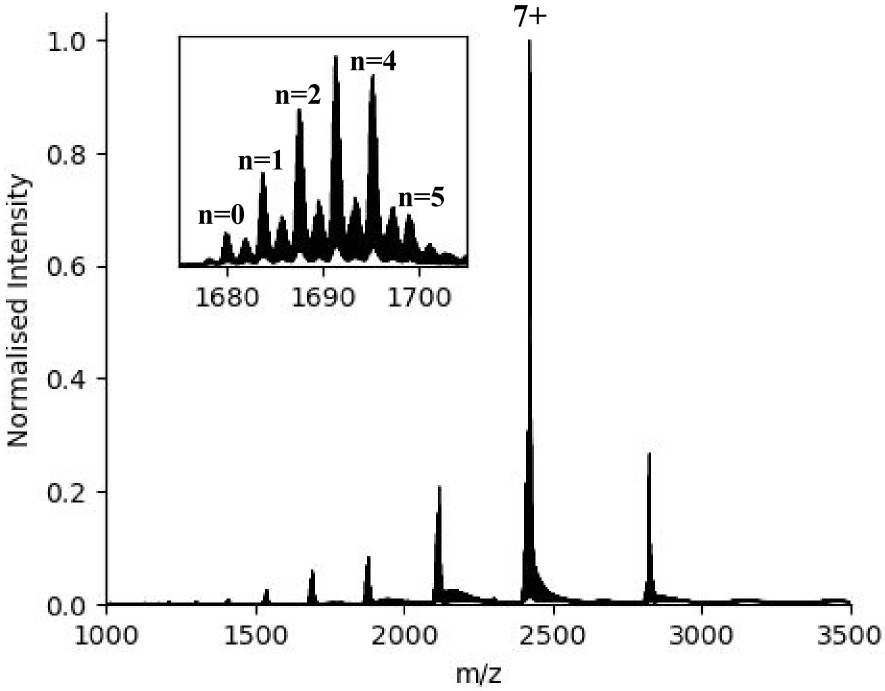

Supplementary Figure 1. Native mass spectrum of calmodulin, with an inset showing the 10+ charge state in which multiple calcium binding states (n=0 to n=5) can be observed.

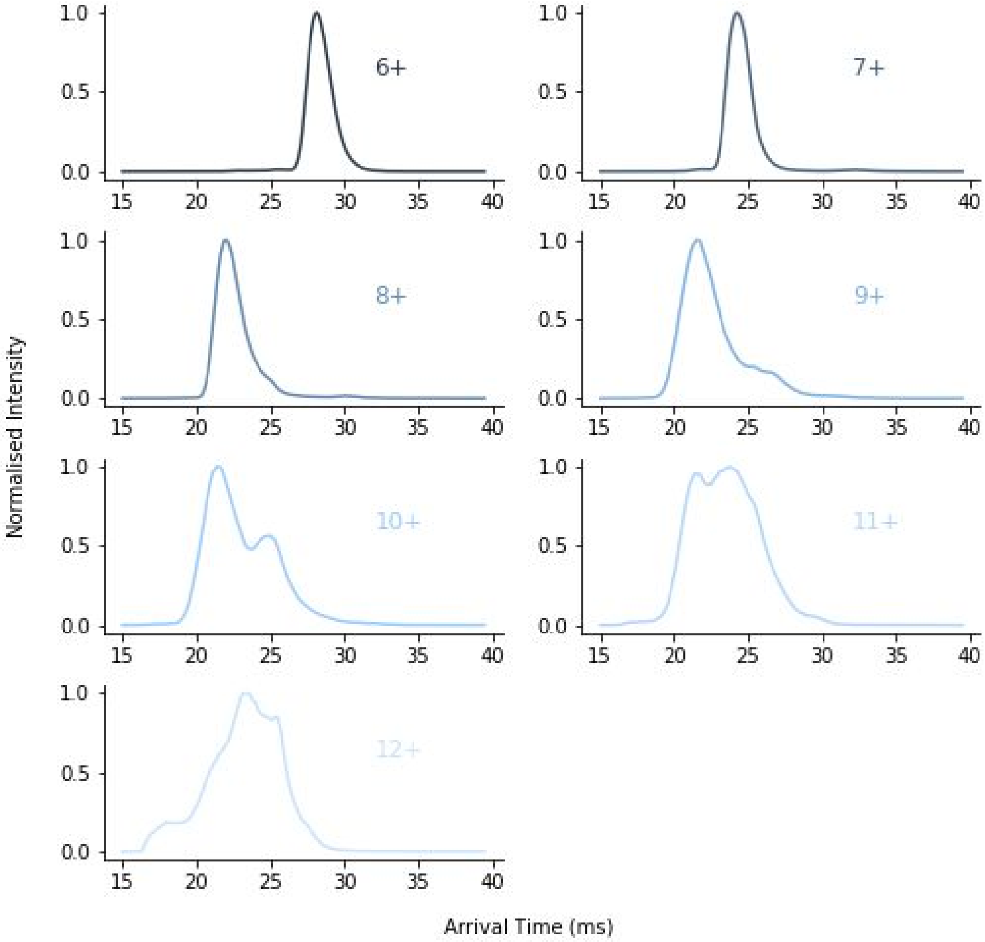

Supplementary Figure 2. Single-pass (5 ms separation) cIM arrival time distributions corresponding to quadrupole isolated charge states of calmodulin from 6+ to 12+.

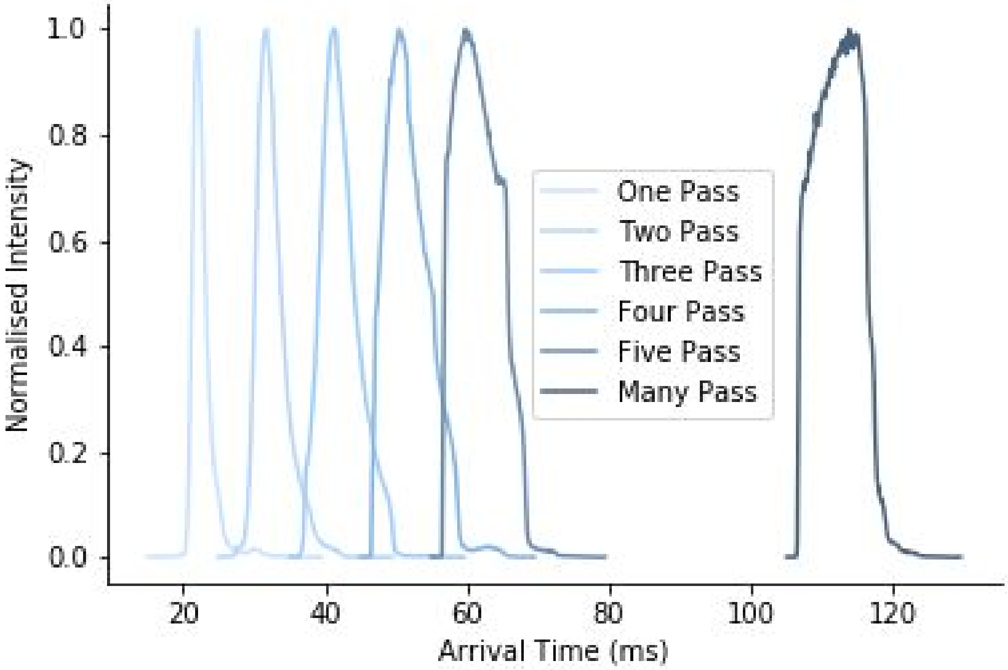

Supplementary Figure 3. Arrival time distributions for the quadrupole-isolated 8+ charge state of calmodulin demonstrating the effects of multiple passes around the cyclic IM racetrack.

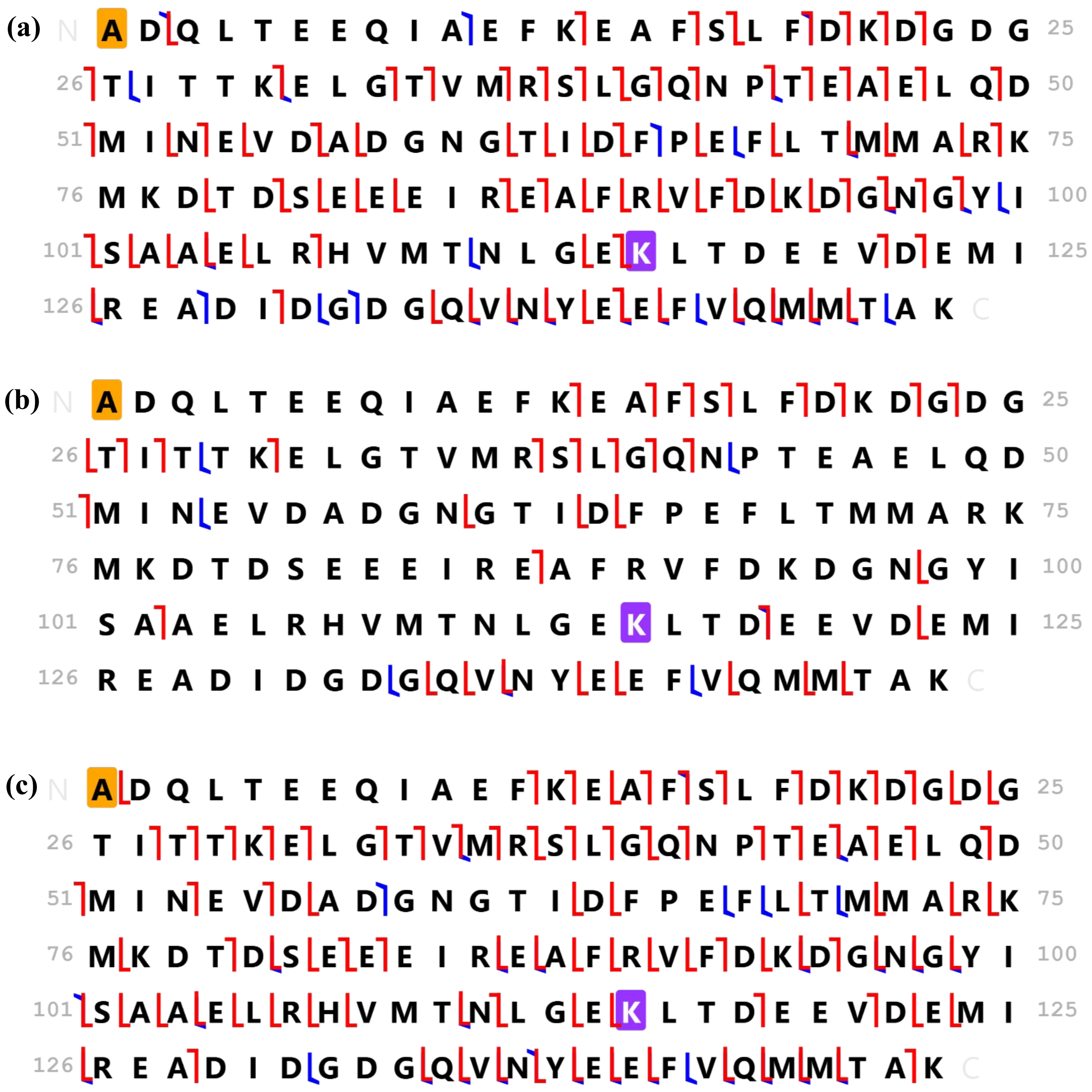

Supplementary Figure 4. Fragment map obtained for the 10+ charge state of calmodulin without conformer-selection (a) mimicking the results obtained from a classic native top-down experiment. Results for the early conformer (b), and late conformer (c) are also shown. The red lines indicate matched *c* and *z* ions, whilst the blue represent *b* and *y* ions. All reported ions are within a 20ppm error tolerance. The matched sequence is bovine calmodulin with *N*-terminal methionine loss, *N*-terminal acetylation (yellow), and K^{115} tri-methylation (purple).

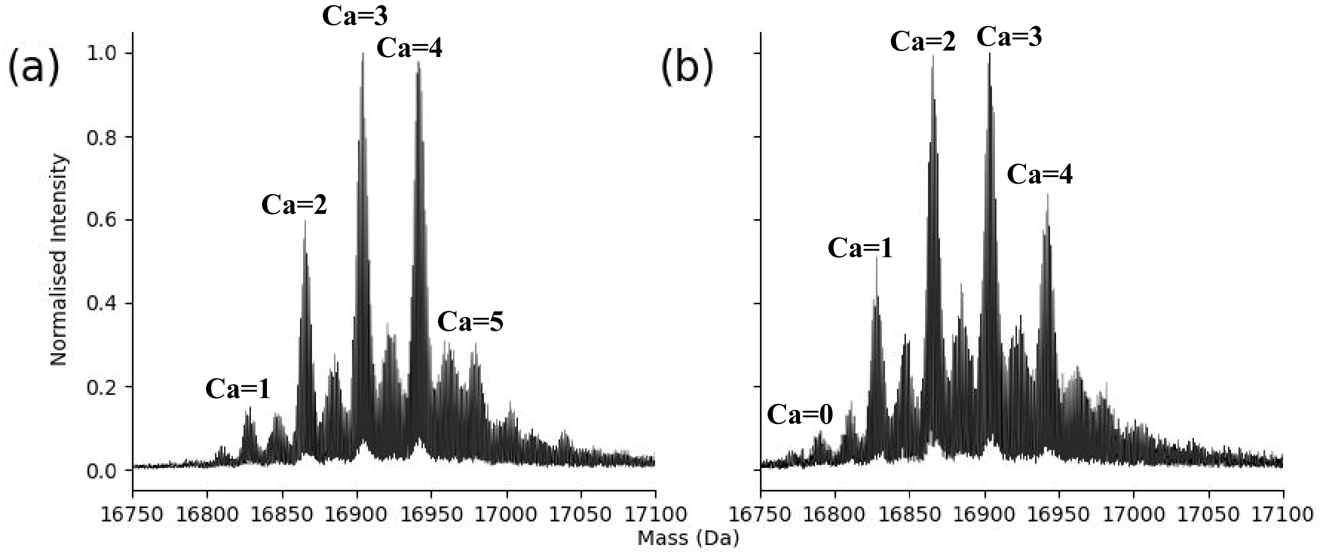

Supplementary Figure 5. Deconvoluted mass measurements for the 10+ charge state of the compact (a) and extended (b) conformers of calmodulin, highlighting their different calcium binding behavior. Calcium binding states n=0 to n=5 are labelled on each spectrum, with the peaks in between these corresponding to the binding of calcium-sodium mixtures.

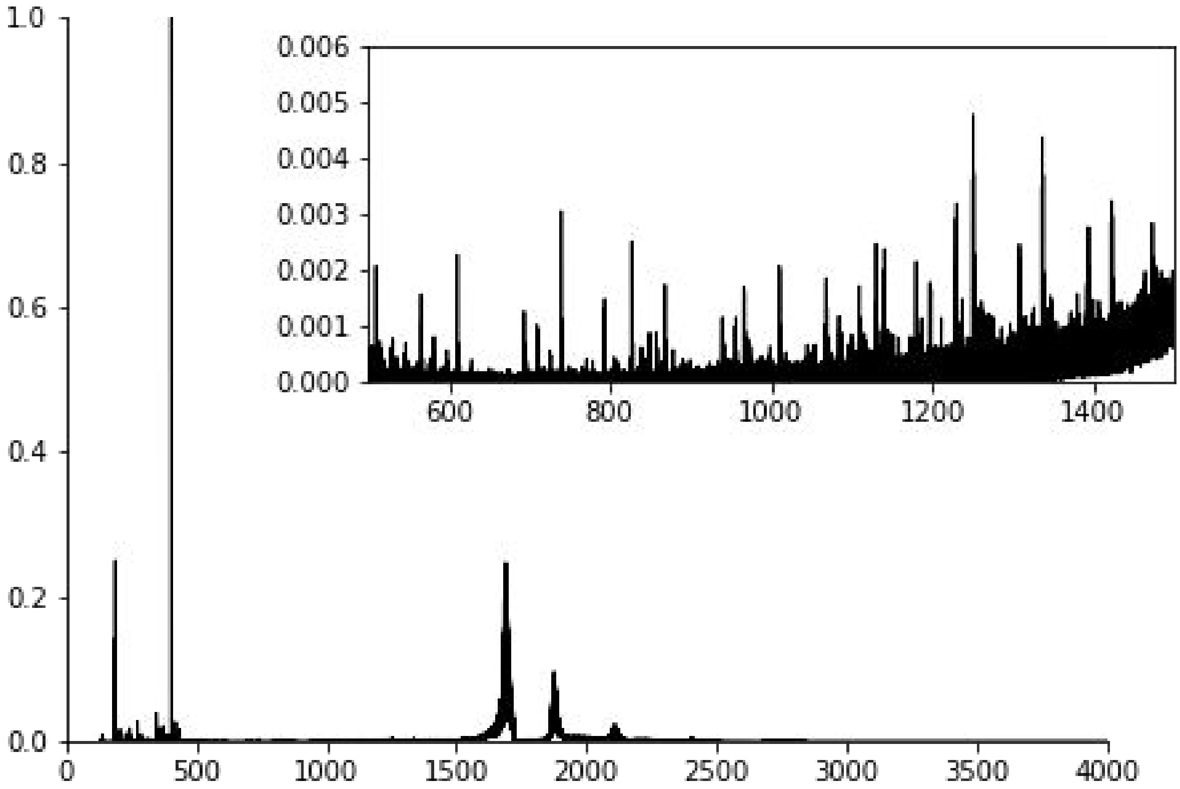

Supplementary Figure 6. ECD spectrum of calmodulin 10+ charge state across the full ATD. Inset shows a zoom of ions between *m/z* 400-1500.

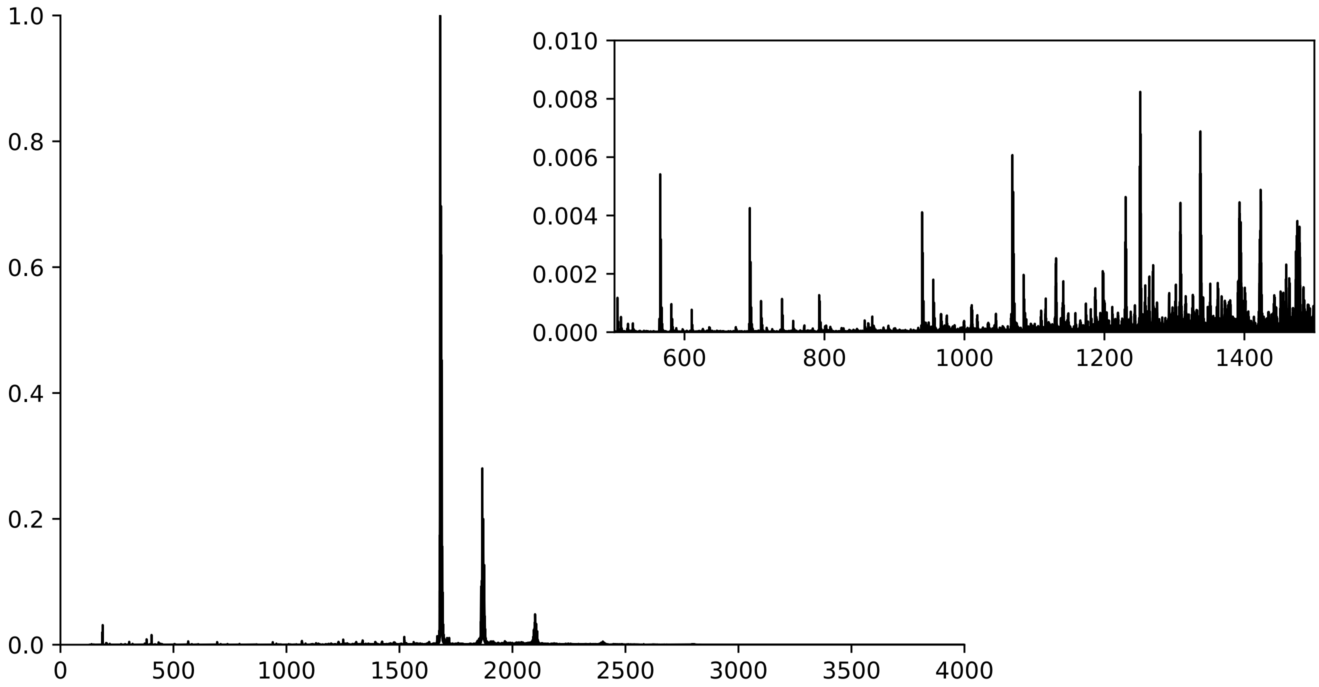

Supplementary Figure 7. ECD spectrum of calmodulin 10+ charge state following isolation of the compact conformational family. Inset shows a zoom of ions between *m/z* 400-1500.

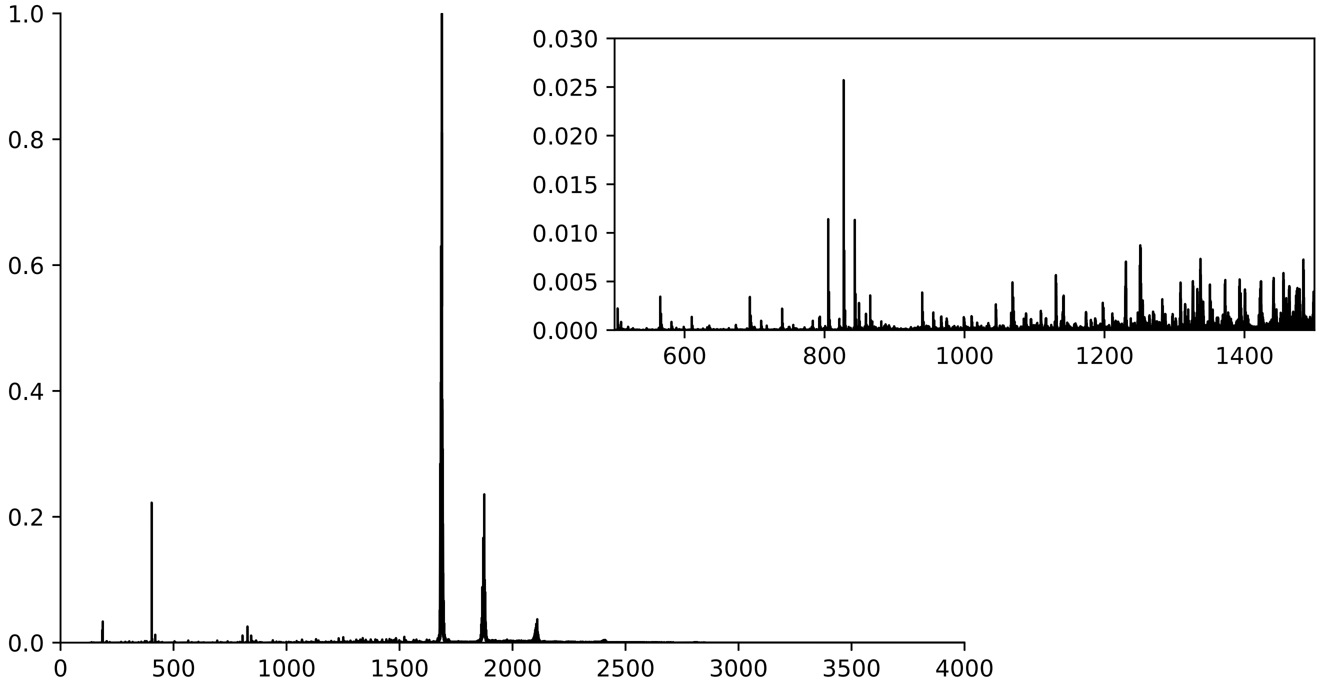

Supplementary Figure 8. ECD spectrum of calmodulin 10+ charge state following isolation of the extended conformational family. Inset shows a zoom of ions between *m/z* 400-1500.

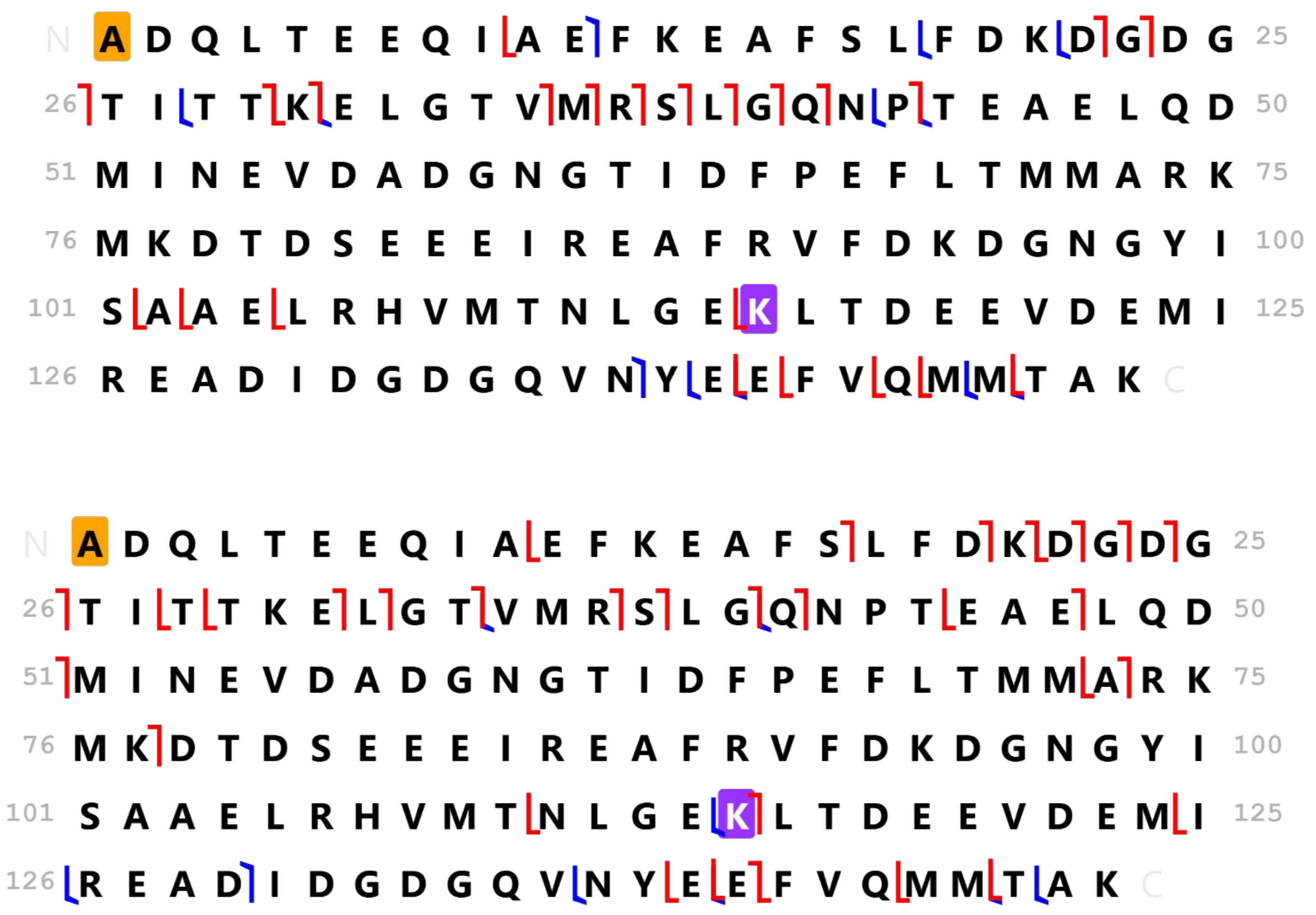

Supplementary Figure 9. Fragment map obtained for the 10+ charge state of calmodulin without conformer-selection but using post-collection extraction of the early conformer (top), and late conformer (bottom). The red lines indicate matched *c* and *z* ions, whilst the blue represent *b* and *y* ions. All reported ions are within a 20ppm error tolerance. The matched sequence is bovine calmodulin with *N*-terminal methionine loss, *N*-terminal acetylation (yellow), and K^{115} tri-methylation (purple).

SUPPLEMENTARY TABLES

Table S2. List of identified fragment ions obtained from ECD of the 10+ charge state of bovine calmodulin with *N*-terminal methionine loss, *N*-terminal acetylation, and K^{115} tri-methylation. Experiment performed without conformational selection, mimicking a classic native top-down experiment.

FRAGMENT ION	THEORETICAL MASS	OBSERVED MASS	MASS DIFFERENCE (PPM)
**B2**	228.10135	228.1024835	4.969427494
**Z146**	16535.75769	16535.91588	9.566716936
**B10**	1140.5566	1140.546014	-9.281842637
**C13**	1561.78886	1561.764924	-15.32631426
**C16**	1908.93697	1908.918504	-9.673691259
**C17**	1995.969	1995.949054	-9.993375088
**Z131**	14784.91634	14785.02654	7.453739379
**B19**	2239.09517	2239.078114	-7.617571199
**C19**	2256.12147	2256.096884	-10.89766983
**C20**	2371.14841	2371.126144	-9.390583392
**C21**	2499.24337	2499.227114	-6.504555369
**C22**	2614.27031	2614.251784	-7.086668433
**C25**	2843.34017	2843.327234	-4.549742945
**Y122**	13852.51626	13852.59085	5.385152543
**C30**	3387.66223	3387.651004	-3.313927457
**Y118**	13409.24188	13409.3916	11.16602374
**C33**	3686.81034	3686.804544	-1.572217268
**C34**	3787.85802	3787.859494	0.389014876
**C36**	4017.96692	4017.950574	-4.068342823
**C37**	4174.06803	4174.055994	-2.883629782
**C38**	4261.10006	4261.088934	-2.611172403
**C39**	4374.18412	4374.170524	-3.108343524
**Z109**	12406.70122	12406.73784	2.951865486
**C40**	4431.20558	4431.201354	-0.953796163
**C41**	4559.26416	4559.252774	-2.497435218
**C43**	4770.35985	4770.355034	-1.009665315
**Y105**	12026.54426	12026.46784	-6.353619564
**C44**	4871.40753	4871.376684	-6.332146652
**C45**	5000.45012	5000.449804	-0.063287679
**C46**	5071.48723	5071.476564	-2.103222664
**C47**	5200.52982	5200.519834	-1.920278746
**C49**	5441.67246	5441.676364	0.717340698
**C50**	5556.6994	5556.686514	-2.319086557
**Z96**	10980.06139	10980.15615	8.630453853
**C53**	5914.86688	5914.837754	-4.924281048
**Z94**	10736.97587	10737.04215	6.173331665
**C56**	6258.00482	6257.935944	-11.00613836
**Z92**	10522.88052	10522.9466	6.279926205
**Z91**	10451.84341	10451.87782	3.292520924

**Z87**	10108.73062	10108.79648	6.515448438
**Z86**	10007.68294	10007.69485	1.190376753
**Z85**	9894.598881	9894.642594	4.417856009
**C64**	7001.3134	7001.198174	-16.45783588
**Z84**	9779.571941	9779.655684	8.563044848
**B65**	7131.35551	7131.315974	-5.544032523
**Z82**	9535.450771	9535.499844	5.146365317
**Y81**	9422.426946	9422.522844	10.17762342
**Z80**	9259.339771	9259.418444	8.496600738
**Z78**	9045.208031	9045.265504	6.353962554
**Y78**	9061.226796	9061.280944	5.975781684
**Z77**	8914.167541	8914.266934	11.14999384
**Z75**	8712.089941	8712.109234	2.214498846
**C74**	8224.89651	8224.915444	2.30197828
**Z70**	8053.731481	8053.763874	4.022099967
**Z68**	7837.656861	7837.725064	8.701951914
**C80**	8943.22848	8943.315884	9.773152203
**Z67**	7750.624831	7750.683234	7.535252241
**Z66**	7621.582241	7621.640984	7.707443319
**Z65**	7492.539651	7492.604304	8.628971769
**Z62**	7094.311891	7094.340854	4.082554245
**C86**	9686.57345	9686.636654	6.52485974
**C87**	9815.61604	9815.677294	6.240416585
**Z60**	6894.232191	6894.260714	4.137213882
**Z59**	6747.163781	6747.194014	4.480832851
**Z58**	6591.062671	6591.095704	5.011773483
**Z57**	6491.994261	6492.018904	3.795892623
**Z56**	6344.925851	6344.940844	2.362976885
**C92**	10435.95949	10436.01031	4.870039327
**Z55**	6229.898911	6229.927004	4.509369016
**Z54**	6101.803951	6101.837964	5.574238929
**C95**	10794.10833	10794.13121	2.120002174
**Z52**	5929.755551	5929.775524	3.368252359
**Y52**	5945.774316	5945.783734	1.583967473
**C96**	10851.12979	10851.16535	3.277403719
**C97**	10965.17272	10965.22596	4.855694888
**Y50**	5774.709926	5774.691414	-3.205717191
**C98**	11022.19418	11022.21195	1.612522229
**Y49**	5611.646596	5611.634194	-2.210061997
**Z48**	5482.543771	5482.571244	5.010979259
**C100**	11298.34157	11298.37081	2.588303155
**Z47**	5395.511741	5395.549444	6.987828946
**Z46**	5324.474631	5324.498514	4.48549665
**Z45**	5253.437521	5253.463484	4.942080879
**Y45**	5269.456286	5269.488514	6.115984529
**Z44**	5124.394931	5124.412454	3.419508714
**C106**	11925.67558	11925.75892	6.988579605

**Y38**	4403.013036	4403.029834	3.815095023
**Z35**	4102.845821	4102.875474	7.227401276
**Z34**	3973.803231	3973.824594	5.375936321
**C114**	12807.08211	12807.1828	7.862332128
**C121**	13663.53629	13663.53459	-0.124205537
**C122**	13778.56323	13778.64093	5.639406071
**Z23**	2629.15497	2629.187354	12.317088
**Y23**	2645.173735	2645.209464	13.50706483
**B128**	14490.88488	14490.83077	-3.73387045
**C130**	14736.02218	14736.10923	5.907490643
**Y17**	1945.854985	1945.866684	6.012027212
**B132**	14891.04428	14891.14956	7.070216914
**Z14**	1700.76636	1700.777944	6.810772715
**Z13**	1572.70778	1572.715654	5.006354785
**Y13**	1588.726545	1588.731304	2.995187016
**Z12**	1473.63937	1473.648084	5.912934533
**Y12**	1489.658135	1489.663714	3.744841175
**Z11**	1359.59644	1359.604614	6.011734717
**Y11**	1375.615205	1375.627284	8.780459155
**Z10**	1196.53311	1196.541464	6.981447526
**Z9**	1067.49052	1067.493384	2.682490445
**Y9**	1083.509285	1083.506364	-2.696300734
**C139**	15713.39482	15713.44736	3.3438295
**Z8**	938.44793	938.4487435	0.866892125
**Y8**	954.466695	954.4615135	-5.428651316
**Y7**	807.398285	807.3963735	-2.367439856
**Z6**	692.31111	692.3094735	-2.363773824
**Y6**	708.329875	708.3260335	-5.423273838
**Z5**	564.25253	564.2490735	-6.125744584
**Y5**	580.271295	580.2667135	-7.89538776
**Z4**	433.21204	433.2080035	-9.317531618
**Y4**	449.230805	449.2266935	-9.152237189
**Z3**	302.17155	302.1667935	-15.74094874
**Y3**	318.190315	318.1849735	-16.78701905
**Y2**	217.142635	217.1434935	3.953774992

Table S3. List of identified fragment ions obtained from ECD of the extended conformer of the 10+ charge state of bovine calmodulin with *N*-terminal methionine loss, *N*-terminal acetylation, and K^{115} tri-methylation.

FRAGMENT ION	THEORETICAL MASS	OBSERVED MASS	MASS DIFFERENCE (PPM)
**B16**	1891.91067	1891.890594	-10.61174145
**B58**	6427.04257	6427.135704	14.49088474
**B100**	11281.31527	11281.15446	-14.25423038
**B137**	15404.2626	15404.30237	2.581942036
**C12**	1433.6939	1433.671074	-15.92143684
**C13**	1561.78886	1561.765674	-14.8460957
**C15**	1761.86856	1761.839604	-16.43508916
**C16**	1908.93697	1908.916404	-10.77377996
**C17**	1995.969	1995.944524	-12.26294941
**C19**	2256.12147	2256.095144	-11.6689049
**C20**	2371.14841	2371.128614	-8.348894062
**C21**	2499.24337	2499.226124	-6.900675255
**C22**	2614.27031	2614.250104	-7.72929517
**C27**	3057.47191	3057.460314	-3.792828592
**C28**	3158.51959	3158.496524	-7.302936146
**C29**	3259.56727	3259.550074	-5.275690132
**C30**	3387.66223	3387.653524	-2.570051643
**C31**	3516.70482	3516.698944	-1.671015107
**C33**	3686.81034	3686.805654	-1.27114401
**C34**	3787.85802	3787.862974	1.307739913
**C35**	3886.92643	3886.893554	-8.458216916
**C36**	4017.96692	4017.950164	-4.170384479
**C37**	4174.06803	4174.055894	-2.907587225
**C38**	4261.10006	4261.086074	-3.282360584
**C39**	4374.18412	4374.165474	-4.262844538
**C40**	4431.20558	4431.195424	-2.292032427
**C41**	4559.26416	4559.248654	-3.401089811
**C43**	4770.35985	4770.359804	-0.00974075
**C44**	4871.40753	4871.356524	-10.47058095
**C45**	5000.45012	5000.454904	0.956620505
**C46**	5071.48723	5071.457464	-5.869376286
**C47**	5200.52982	5200.518284	-2.218325301
**C49**	5441.67246	5441.670844	-0.297053322
**C50**	5556.6994	5556.691034	-1.505654036
**C53**	5914.86688	5914.849804	-2.887041624
**C55**	6142.97788	6142.969304	-1.396141586
**C79**	8828.20154	8828.241284	4.501883304
**C82**	9159.3031	9159.354574	5.619808905
**C83**	9288.34569	9288.400904	5.944388265
**C92**	10435.95949	10436.00404	4.269232088
**C95**	10794.10833	10794.0748	-3.105996887

**C111**	12507.934	12508.02504	7.278862611
**C118**	13306.3827	13306.46192	5.953752791
**C121**	13663.53629	13663.2853	-18.36911628
**C128**	14507.91118	14507.92854	1.196789319
**C147**	16650.79858	16650.85859	3.6042063
**Y113**	12909.97768	12909.95153	-2.024952136
**Y103**	11796.45399	11796.50161	4.037477126
**Y81**	9422.426946	9422.471224	4.699204714
**Y80**	9275.358536	9275.322924	-3.839429682
**Y78**	9061.226796	9061.243564	1.850512464
**Y68**	7853.675626	7853.740794	8.297759702
**Y62**	7110.330656	7110.353884	3.266783817
**Y61**	6981.288066	6981.216364	-10.27060998
**Y54**	6117.822716	6117.837704	2.449876993
**Y52**	5945.774316	5945.772544	-0.298041396
**Y51**	5831.731386	5831.758454	4.641488321
**Y50**	5774.709926	5774.697074	-2.225581379
**Y45**	5269.456286	5269.491834	6.746030557
**Y38**	4403.013036	4403.031034	4.087635665
**Y23**	2645.173735	2645.207544	12.78121458
**Y17**	1945.854985	1945.872094	8.792296061
**Y14**	1716.785125	1716.787014	1.100040473
**Y13**	1588.726545	1588.734884	5.248564107
**Y12**	1489.658135	1489.656834	-0.873668159
**Y10**	1212.551875	1212.549284	-2.137200835
**Y9**	1083.509285	1083.510384	1.013865903
**Y8**	954.466695	954.4618935	-5.030523228
**Y7**	807.398285	807.3954335	-3.531673194
**Y6**	708.329875	708.3264135	-4.886800631
**Y5**	580.271295	580.2667735	-7.791987849
**Y4**	449.230805	449.2254435	-11.93477121
**Y3**	318.190315	318.1854035	-15.43562657
**Z147**	16650.78463	16650.85859	4.442007676
**Z134**	15090.05389	15090.11986	4.371946821
**Z125**	14109.59357	14109.67934	6.079049174
**Z124**	13994.56663	13994.66874	7.296611309
**Z111**	12606.81731	12606.91114	7.443029499
**Z108**	12349.67976	12349.9055	18.27925238
**Z92**	10522.88052	10522.97883	9.342775767
**Z85**	9894.598881	9894.639934	4.149022474
**Z84**	9779.571941	9779.634564	6.403441123
**Z79**	9146.255711	9146.216934	-4.239667915
**Z77**	8914.167541	8914.271874	11.70416785
**Z75**	8712.089941	8712.116534	3.052414897
**Z74**	8555.988831	8556.048994	7.031672705
**Z72**	8296.853381	8296.920994	8.14922357
**Z68**	7837.656861	7837.693724	4.703307861

**Z67**	7750.624831	7750.667744	5.536703693
**Z66**	7621.582241	7621.627914	5.992576302
**Z62**	7094.311891	7094.337844	3.658270671
**Z61**	6965.269301	6965.289714	2.930671054
**Z60**	6894.232191	6894.271704	5.731300024
**Z59**	6747.163781	6747.184134	3.016513869
**Z58**	6591.062671	6591.088214	3.87538617
**Z57**	6491.994261	6492.039454	6.961329802
**Z55**	6229.898911	6229.936334	6.006985612
**Z54**	6101.803951	6101.839414	5.811873572
**Z52**	5929.755551	5929.772024	2.778008803
**Z51**	5815.712621	5815.734494	3.761003087
**Z50**	5758.691161	5758.710564	3.369326915
**Z48**	5482.543771	5482.567544	4.336110046
**Z47**	5395.511741	5395.541764	5.564423647
**Z46**	5324.474631	5324.499834	4.733408433
**Z45**	5253.437521	5253.465264	5.280906647
**Z44**	5124.394931	5124.419434	4.781620748
**Z43**	5011.310871	5011.332054	4.227020368
**Z42**	4855.209761	4855.237874	5.790257168
**Z41**	4718.150851	4718.157284	1.363439475
**Z38**	4386.994271	4387.032814	8.785722238
**Z37**	4272.951341	4272.972274	4.898935525
**Z35**	4102.845821	4102.869504	5.772313695
**Z34**	3973.803231	3973.827124	6.012605993
**Z26**	3002.32211	3002.342764	6.879186298
**Z25**	2873.27952	2873.300904	7.442204273
**Z23**	2629.15497	2629.180624	9.757330174
**Z14**	1700.76636	1700.779604	7.786803309
**Z13**	1572.70778	1572.718344	6.716780609
**Z12**	1473.63937	1473.649244	6.700101342
**Z11**	1359.59644	1359.603344	5.077634007
**Z10**	1196.53311	1196.542444	7.80048044
**Z9**	1067.49052	1067.493044	2.363986446
**Z8**	938.44793	938.4486135	0.728365526
**Z6**	692.31111	692.3102135	-1.294890212
**Z5**	564.25253	564.2494135	-5.523177503
**Z4**	433.21204	433.2063135	-13.21862356
**Z3**	302.17155	302.1662935	-17.39563794

Table S4. List of identified fragment ions obtained from ECD of the compact conformer of the 10+ charge state of bovine calmodulin with *N*-terminal methionine loss, *N*-terminal acetylation, and K^{115} tri-methylation.

FRAGMENT ION	THEORETICAL MASS	OBSERVED MASS	MASS DIFFERENCE (PPM)
**B118**	13289.3564	13289.28217	-5.585453849
**C13**	1561.78886	1561.764774	-15.42235797
**C15**	1761.86856	1761.851494	-9.686572124
**C16**	1908.93697	1908.914034	-12.01530865
**C17**	1995.969	1995.950704	-9.166708942
**C19**	2256.12147	2256.099074	-9.926977416
**C20**	2371.14841	2371.128834	-8.256112016
**C22**	2614.27031	2614.253294	-6.509069401
**C23**	2671.29177	2671.270754	-7.867529529
**C26**	2944.38785	2944.369764	-6.142691725
**C27**	3057.47191	3057.476894	1.629952218
**C30**	3387.66223	3387.634374	-8.222917454
**C37**	4174.06803	4174.045804	-5.324893299
**C38**	4261.10006	4261.104914	1.13903289
**C39**	4374.18412	4374.173754	-2.369920103
**C40**	4431.20558	4431.218274	2.864577797
**C41**	4559.26416	4559.264384	0.049028332
**C50**	5556.6994	5556.673564	-4.649606722
**C87**	9815.61604	9815.516634	-10.12737932
**C102**	11456.41071	11456.27192	-12.11430616
**C118**	13306.3827	13306.55491	12.94212838
**Y7**	807.398285	807.3970535	-1.525228506
**Y12**	1489.658135	1489.681884	15.94227062
**Y15**	1773.806585	1773.818384	6.651533048
**Y95**	10882.03723	10882.25248	19.78103076
**Y106**	12123.59702	12123.48002	-9.649948502
**Y120**	13638.38452	13638.50004	8.470791611
**Z3**	302.17155	302.1736435	6.928293282
**Z4**	433.21204	433.2063835	-13.05703987
**Z6**	692.31111	692.3130335	2.778423013
**Z9**	1067.49052	1067.494794	4.003345266
**Z10**	1196.53311	1196.534884	1.48222653
**Z12**	1473.63937	1473.651794	8.430511137
**Z13**	1572.70778	1572.719374	7.371702021
**Z14**	1700.76636	1700.779114	7.498697893
**Z26**	3002.32211	3002.338964	5.613499319
**Z51**	5815.712621	5815.749524	6.34538113
**Z84**	9779.571941	9779.652094	8.195953117
**Z85**	9894.598881	9894.548314	-5.110574717
**Z88**	10165.75208	10165.89352	13.91366935
**Z123**	13937.54517	13937.65405	7.812201633

Table S5. List of identified fragment ions obtained from ECD of the 7+ charge state of bovine calmodulin with *N*-terminal methionine loss, *N*-terminal acetylation, and K^{115} tri-methylation. A single conformer is observed in the ion mobility and selected for isolation.

FRAGMENT ION	THEORETICAL MASS	OBSERVED MASS	MASS DIFFERENCE (PPM)
Z2	201.12387	201.1202935	-17.78240882
B2	228.10135	228.1035635	9.704164925
Z3	302.17155	302.1689835	-8.493410051
Y3	318.190315	318.1876835	-8.270103633
Z4	433.21204	433.2110635	-2.254016024
Y4	449.230805	449.2281535	-5.902237448
Z5	564.25253	564.2518835	-1.145704885
Y5	580.271295	580.2635435	-13.3583497
Z6	692.31111	692.3118635	1.088431356
Y6	708.329875	708.3238735	-8.472700491
Z7	791.37952	791.3802835	0.964812837
Y7	807.398285	807.3925435	-7.111071434
Z8	938.44793	938.4517235	4.042348008
Y8	954.466695	954.4625835	-4.307606438
Z9	1067.49052	1067.496784	5.86753044
Y9	1083.509285	1083.505114	-3.849959514
Z10	1196.53311	1196.541444	6.964732568
Y10	1212.551875	1212.544234	-6.301971105
Z11	1359.59644	1359.603854	5.452745316
Y11	1375.615205	1375.622934	5.618237638
Z12	1473.63937	1473.651294	8.091215098
Y12	1489.658135	1489.653574	-3.062089732
C13	1561.78886	1561.772834	-10.26160917
Z13	1572.70778	1572.719684	7.56881429
Y13	1588.726545	1588.723504	-1.914405528
C14	1690.83145	1690.813424	-10.66130328
Z14	1700.76636	1700.776454	5.934697062
Y14	1716.785125	1716.793984	5.159954493
C15	1761.86856	1761.854604	-7.92140072
Y15	1773.806585	1773.812074	3.094211718
Z16	1872.81476	1872.829214	7.717545498
C16	1908.93697	1908.921474	-8.117851518
Y17	1945.854985	1945.865884	5.600896883
C21	2499.24337	2499.229874	-5.40022114
C22	2614.27031	2614.248234	-8.444599931
C23	2671.29177	2671.284684	-2.652823986
C26	2944.38785	2944.355744	-10.90429268
C30	3387.66223	3387.641234	-6.197922182
C34	3787.85802	3787.846434	-3.058844027
C35	3886.92643	3886.932334	1.518817818
Z34	3973.803231	3973.828194	6.281869451

**C36**	4017.96692	4017.962074	-1.206198801
**C37**	4174.06803	4174.067444	-0.140502473
**C38**	4261.10006	4261.085964	-3.308175514
**C39**	4374.18412	4374.191344	1.651401249
**Z38**	4386.994271	4387.043514	11.22474981
**Y38**	4403.013036	4403.024714	2.65225495
**C40**	4431.20558	4431.189974	-3.521946026
**Z39**	4488.041951	4488.005394	-8.145442329
**C41**	4559.26416	4559.272054	1.731317345
**Z41**	4718.150851	4718.169084	3.864419282
**C43**	4770.35985	4770.322984	-7.728236033
**Z42**	4855.209761	4855.234884	5.174423837
**C44**	4871.40753	4871.349404	-11.93217084
**C45**	5000.45012	5000.453674	0.710642649
**C46**	5071.48723	5071.490324	0.609985391
**B50**	5539.6731	5539.722094	8.844119903
**B56**	6240.97852	6241.057364	12.63320053
**Y70**	8069.750246	8069.756994	0.836198509
**C105**	11769.57447	11769.54915	-2.151009532
**Y108**	12365.69853	12365.69491	-0.292105364
**B122**	13761.53693	13761.45324	-6.081231136
**Z127**	14281.64197	14281.78714	10.16500158
**C132**	14908.07058	14908.20034	8.704205715
**B133**	15006.07122	15006.09588	1.643528995
**Y134**	15106.07266	15106.21855	9.658229274
**C135**	15208.17756	15208.19015	0.828035647
**Z135**	15219.09648	15219.28485	12.37740449
**C136**	15307.24597	15307.32321	5.046166586
**Y136**	15363.21021	15363.39822	12.2381918
**Z142**	16064.52478	16064.77739	15.72489175
**B143**	16199.60651	16199.61528	0.541551002
**B145**	16461.68749	16461.39991	-17.46947796

Table S6. List of identified fragment ions obtained from ECD of the 7+ charge state of bovine calmodulin with *N*-terminal methionine loss, *N*-terminal acetylation, and K^{115} tri-methylation, in complex with on equivalent of the peptide melittin.

FRAGMENT ION	THEORETICAL MASS	OBSERVED MASS	MASS DIFFERENCE (PPM)
**B143**	16199.60651	16199.68577	4.892891262
**C94**	10679.08139	10679.12802	4.366811285
**Y4**	449.230805	449.2261835	-10.28751107
**Y8**	954.466695	954.4621635	-4.747642745
**Y119**	13537.33684	13537.42675	6.642215838
**Y129**	14540.78264	14541.01286	15.83325457
**Y135**	15235.11525	15235.24253	8.354903202
**Z3**	302.17155	302.1699235	-5.382594357
**Z4**	433.21204	433.2100135	-4.677771375
**Z5**	564.25253	564.2488135	-6.586531176
**Z6**	692.31111	692.3082335	-4.154876093
**Z7**	791.37952	791.3728335	-8.449128024
**Z8**	938.44793	938.4467135	-1.296253997
**Z9**	1067.49052	1067.489644	-0.821053549
**Z25**	2873.27952	2873.251674	-9.69152729
**Z118**	13393.22311	13392.96949	-18.93622504

Table S7. List of identified fragment ions obtained from ECD of the 7+ charge state of bovine calmodulin with *N*-terminal methionine loss, *N*-terminal acetylation, and K^{115} tri-methylation, in complex with on equivalent of the 290-309 peptide of CaMKII.

FRAGMENT ION	THEORETICAL MASS	OBSERVED MASS	MASS DIFFERENCE (PPM)
**B27**	3040.44561	3040.426944	-6.139385233
**B133**	15006.07122	15006.10269	2.097345313
**Y3**	318.190315	318.1885735	-5.473035469
**Y4**	449.230805	449.2269635	-8.55120984
**Y5**	580.271295	580.2622335	-15.61591441
**Y8**	954.466695	954.4561635	-11.03387571
**Y9**	1083.509285	1083.503934	-4.939013402
**Z3**	302.17155	302.1679935	-11.76969467
**Z4**	433.21204	433.2074935	-10.49478422
**Z5**	564.25253	564.2500335	-4.424378709
**Z6**	692.31111	692.3105335	-0.832670271
**Z7**	791.37952	791.3765835	-3.710567188
**Z8**	938.44793	938.4487435	0.866892125
**Z9**	1067.49052	1067.493344	2.645019386
**Z10**	1196.53311	1196.536964	3.220582103
**Z11**	1359.59644	1359.594084	-1.73321054
**Z12**	1473.63937	1473.648984	6.523667402
**Z13**	1572.70778	1572.714794	4.459527198
**Z14**	1700.76636	1700.766954	0.348979809
**Z138**	15623.30244	15623.4175	7.364826584
**Z139**	15694.33955	15694.58374	15.55929846
**Z145**	16407.69911	16407.7242	1.52933772
